# Factor B as a therapeutic target for the treatment of complement-mediated diseases

**DOI:** 10.3389/fimmu.2025.1537974

**Published:** 2025-02-14

**Authors:** David Kavanagh, Jonathan Barratt, Anna Schubart, Nicholas J. A. Webb, Matthias Meier, Fadi Fakhouri

**Affiliations:** ^1^ National Renal Complement Therapeutics Centre, Newcastle University, Newcastle-upon-Tyne, United Kingdom; ^2^ Department of Cardiovascular Sciences, University of Leicester, The John Walls Renal Unit, University Hospitals of Leicester NHS Trust, Leicester, United Kingdom; ^3^ Department of Immunology, Novartis BioMedical Research, Basel, Switzerland; ^4^ Novartis Pharma AG, Basel, Switzerland; ^5^ Service of Nephrology and Hypertension, Centre Hospitalier Universitaire Vaudois, UNIL, Lausanne, Switzerland

**Keywords:** complement, alternative pathway, factor B, therapeutic target, inhibitors, pharmacological treatment

## Abstract

The complement system, consisting of three initiating pathways—classical, lectin and alternative, is an important part of innate immunity. Dysregulation of the complement system is implicated in the pathogenesis of several autoimmune and inflammatory diseases. Therapeutic inhibition of the complement system has been recognized as a viable approach to drug development and has been successful with the approval of a small number of complement inhibitors for diseases such as paroxysmal nocturnal hemoglobinuria, atypical hemolytic uremic syndrome, neuromyelitis optica, myasthenia gravis and geographic atrophy. More recently, therapies selectively targeting the alternative pathway (AP), which drives the amplification of the complement responses, are being evaluated for these complement-mediated diseases. Complement Factor B, a serine protease, is a unique component of the AP that is essential for the catalytic activity of AP C3 convertase and AP C5 convertase. Inhibition of Factor B blocks the activity of the alternative pathway and the amplification loop, and subsequent generation of the membrane attack complex downstream; however, it has no effect on the initial activation mediated by the classical and lectin complement pathways. Therefore, Factor B is an attractive target for diseases in which the AP is overactivated. In this review, we provide an overview of Factor B and its critical role in the AP, discuss the benefit-risk of Factor B inhibition as a targeted therapeutic strategy, and describe the various Factor B inhibitors that are approved and/or in clinical development.

## 1 Introduction

The complement system is a central player in innate immunity, which serves as the first line of defense against invading pathogens and altered host cells, modulates adaptive immunity, and contributes to the maintenance of tissue homeostasis (1, 2). Its activities are mediated by sequentially activated serine proteases generating effector molecules that trigger inflammatory responses, enable opsonization, and promote phagocytosis and lysis of pathogens (1, 3–5). The balance between the activation and regulation of the complement system ensures that infectious pathogens and damaged cells are destroyed or removed, while leaving healthy host cells intact (1, 4). However, failure of regulatory mechanisms can lead to aberrant activation of the complement system, which underlies the pathogenesis of several inflammatory and autoimmune disorders and forms the rationale for the development of targeted complement therapies (1, 3, 4, 6, 7). In this review, we outline the role of Factor B (FB), a key protease of the alternative pathway (AP) of the complement system in health and disease, as an attractive therapeutic target for a number of complement-mediated diseases.

### 1.1 Overview of the complement system

**Figure 1:**
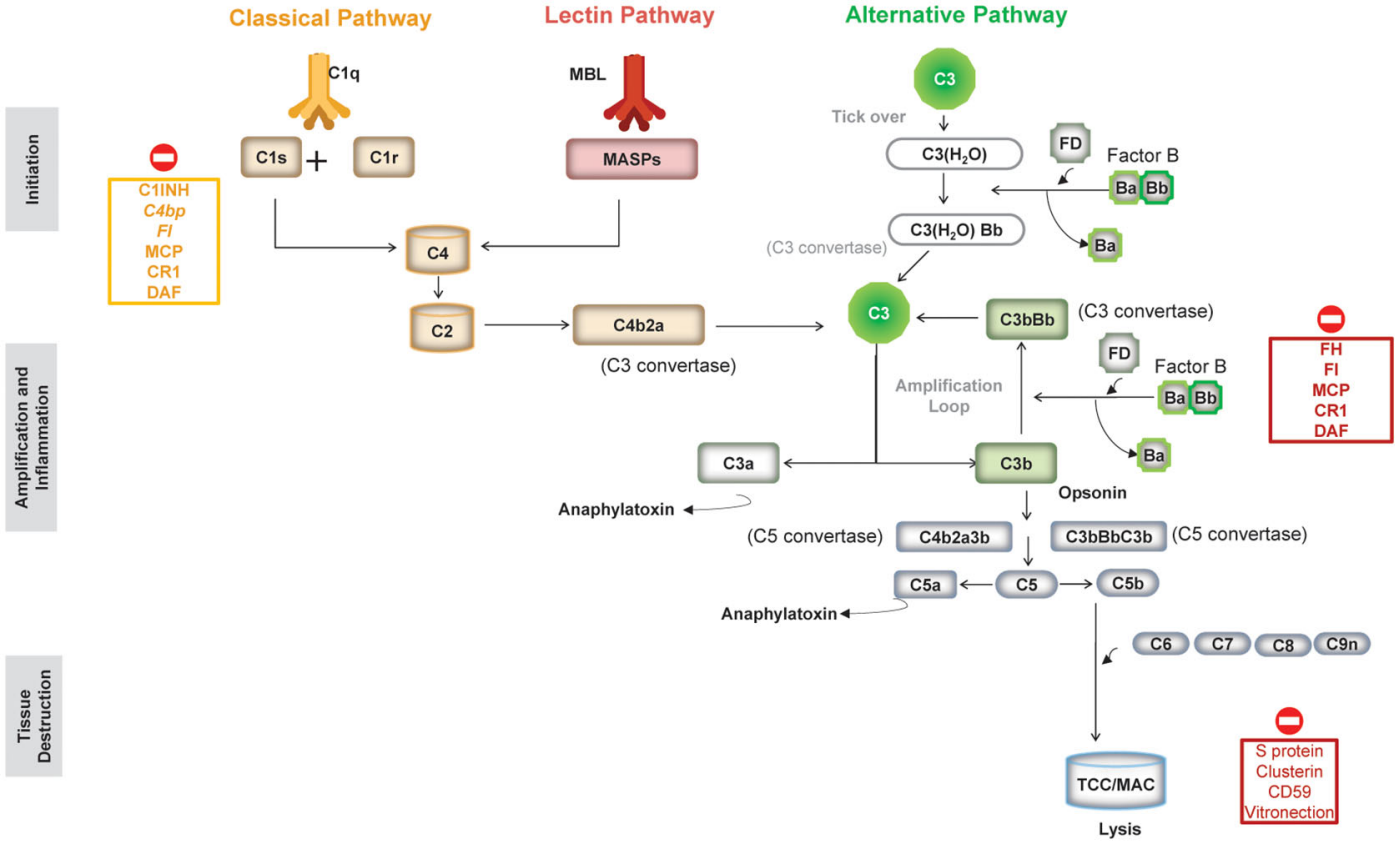
An overview of the three pathways of the complement system and complement regulators. (Figure adapted from Morgan BP et al. Front Cell Neurosci. 2021;14:600656 (https://doi.org/10.3389/fncel.2020.600656). Copyright 2021 Morgan, Gommerman and Ramaglia, CC BY (101). C1-INH, serum protein C1 inhibitor; C4Bp, complement inhibitor C4b binding protein; CR1, complement receptor type 1; DAF, decay accelerating factor (CD55); FI, factor I; FH, factor H; MAC, membrane attack complex; MBL, mannose-binding lectin; MCP, membrane cofactor protein (CD46); TCC, terminal complement complex.

Composed of more than 50 plasma and cell surface-bound proteins and regulators, the complement system can be activated through three distinct interconnected pathways: the classical pathway (CP), the lectin pathway (LP), and the AP (1, 4) (**Figure 1**).

The CP is activated by antigen–antibody complexes and the LP by conserved carbohydrate moieties present on pathogen surfaces, whereas the AP, evolutionarily the oldest, is constitutively active at low levels through a process of slow, spontaneous hydrolysis of C3 called “tick-over” (1). All three pathways converge at the cleavage of the central complement protein C3 by C3 convertases into C3a, an anaphylatoxin, and C3b, which is an opsonin that also triggers further activation of the AP acting as an amplification loop (1). Downstream of C3 convertase activation, the three pathways share the same sequence of events: C5 convertase formation, culminating in the assembly of the membrane attack complex (MAC), thus mediating cell lysis (**Figure 1**) (1).

#### 1.1.1 AP and amplification loop

The AP is driven by two serine proteases that control activation (Factor D [FD]) and activity (Factor B [FB]) of the pathway. These two factors have distinct characteristics. The mannose-binding lectin-associated serine protease-3 (MASP-3), as a pro-FD activator, also plays an important role in AP activation.

FB is mainly produced in hepatocytes, found in high levels in serum (2-3 µM), and has a low clearance rate (plasma half-life 30-70 hours) (3). Factor D is largely produced in adipocytes, its latent proenzyme form (Pro-FD) is continuously converted into its active form (the predominant form that circulates in plasma) by MASP-3 (8, 9). FD is present in the serum at 2 µg/mL and has a rapid clearance rate (plasma half-life ~1 hours) (3, 10). While FD has long been considered rate-limiting for AP activation, it was recently shown that low levels of FD (<1% of serum levels) were sufficient for normal AP activation (11). Since FD is cleared through the kidneys, its levels can increase in renally impaired patients by 10-fold (10).

As shown in **Figure 1**, the AP is continuously active through spontaneous hydrolysis of a labile thioester bond in C3 [to form C3(H_2_O)] that induces a conformational change to expose the binding site for FB. The FB in this complex [C3(H_2_O)FB] is then cleaved by FD to form C3(H_2_O)Bb that acts as the initial fluid-phase C3 convertase of the AP to cleave C3 into C3a and C3b (1, 3).

FB can also bind to C3b, generated from CP/LP or AP C3 convertases, to form the AP C3 convertase C3bBb (5, 12). Since C3b is both a result of the AP C3 convertase activity and a component of the convertase itself, this creates a positive feedback loop that amplifies the initial complement response rapidly, called amplification loop (1, 3, 5). With continued activation of the AP C3 convertase and increasing amounts of C3b in the vicinity of the C3bBb convertase, binding of an additional molecule of C3b to the C3 convertase switches the specificity of the convertase from cleaving C3 to C5 (into C5a and C5b) to form the AP C5 convertase (C3BbC3b), culminating in the formation of the MAC.

The amplification loop enables a rapid scale-up of the initial complement response, not only of the AP but also of those initiated by the CP/LP. Indeed, the amplification loop of the AP may contribute up to 80% of the initial complement response of the CP and LP (13, 14). However, the extent of the contribution by the AP is likely to depend on the strength of the trigger, and amplification may be redundant in the presence of strong LP/CP activation for example after vaccination against encapsulated bacteria, which can result in antibody titers that trigger bacterial clearance *in vitro* in the absence of AP activity (15–19). Not surprisingly, given its potential for rapidly producing copious amounts of C3b, a “runaway” activation of the amplification loop can result in uncontrolled inflammation and mediate the pathogenesis of several complement-mediated diseases (1, 3, 5).

### 1.2 AP in inflammatory and autoimmune diseases

The AP, and in particular the amplification loop is tightly regulated as its uncontrolled activation can induce excessive inflammation and tissue damage. The dysregulation of the AP is implicated in the pathogenesis of several distinct diseases (7, 20, 21). Here, we discuss the prototypical complement-mediated diseases involving the AP.

The pathogenesis of some diseases, such as atypical hemolytic uremic syndrome (aHUS), C3 glomerulopathy (C3G), but also paroxysmal nocturnal hemoglobinuria (PNH), and age-related macular degeneration (AMD), is a direct result of dysregulation of the AP, either due to pathogenic variants in genes encoding AP components and/or their regulators or due to acquired factors such as autoantibodies against components or regulators of the AP (**Supplementary Table S1**). In others, complement activation occurs downstream of disease-specific triggers, such as autoantibodies and immune complexes, which are the key drivers of pathophysiology and tissue injury in these diseases (e.g., systemic lupus erythematosus, rheumatoid arthritis, immunoglobulin A nephropathy [IgAN], anti-neutrophil cytoplasmic autoantibody-associated vasculitis [AAV], and lupus nephritis [LN]) (**Supplementary Table S1**). Irrespective of the initial trigger, as noted earlier, the AP and its amplification loop may play a central role in turning up the magnitude of any initial complement response and, thus, play a key role in exacerbating tissue injury in all these complement-mediated diseases (6, 21).

Although most known complement-mediated diseases are rare or ultra-rare, and many progress slowly, collectively they impose a large burden, with a substantial proportion of patients continuing to remain at risk of disease progression and/or disease relapse despite optimal treatment with current standard of care (SoC). In many diseases, where the complement system mediates or contributes to the pathophysiology of the disease, there are no approved therapies that specifically target the complement pathway (**Supplementary Table S1**). The current management of these rare kidney diseases involving complement dysregulation relies on the use of broad, non-specific immunosuppressive and anti-inflammatory therapies such as corticosteroids, mycophenolate acids, calcineurin inhibitors, and cyclophosphamide. All of these therapies individually have a relatively high toxicity level, limiting their benefit-risk ratio; most are used in combination. A few regimens, which can be nephrotoxic, are associated with poor tolerability and several serious adverse events, including increased risk of serious infections. Moreover, they impose an additional burden on patients and their quality of life (QoL) (22). Furthermore, a majority of these drugs are not approved for most of the diseases/indications and are used off-label, as a rescue therapy, based on limited evidence (22).

PNH and aHUS, and to some extent AAV, have been success stories where complement inhibition has demonstrated efficacy and has emerged as the novel SoC (23–26).The C5 inhibitors eculizumab and ravulizumab (approved for PNH, aHUS, myasthenia gravis, and neuromyelitis optica spectrum disorder) are monoclonal antibodies that block the cleavage of the terminal complement protein C5 to C5a and C5b through C5 convertase, thereby preventing the formation of MAC, regardless of the initial pathway. While anti-C5 therapies can potently control intravascular hemolysis and the associated risk of thrombosis, the most severe complication of PNH, they exposed an additional pathomechanism (e.g. persistent anemia caused by extravascular hemolysis) necessitating more upstream complement-targeting therapies that are safe and well tolerated. Pegcetacoplan, a pegylated cyclic peptide inhibiting C3 cleavage prevents both intra-and extravascular hemolysis and is administered twice weekly by subcutaneous injection. Iptacopan, the first oral FB inhibitor that specifically targets the AP with high potency even during many complement amplifying conditions, has recently received FDA approval for treatment of PNH and IgAN (27–29). Iptacopan improved hemoglobin to near-normal levels and led to blood transfusion avoidance in the majority of treatment-naïve PNH patients and in PNH patients who had residual anemia despite anti-C5 therapy (27, 28).

## 2 Factor B

FB is an abundant plasma protein with circulating concentrations ranging from 0.2 to 0.4 mg/mL. FB is unique to the AP and provides the active protease site of the AP C3 convertase that is responsible for AP activity. The gene encoding FB (*CFB*) resides within the major histocompatibility complex class III region gene cluster on chromosome 6 (6p21.33), in proximity to its CP/LP homologue C2 (30). Like most other members of the complement system, FB is primarily, but not exclusively, synthesized in the liver. Low FB expression is observed in other cell types including renal tubular cells, monocytes, adipocytes, dendritic cells, and fibroblasts, and tissue-specific expression may be significantly upregulated during inflammation (30–33). Various proinflammatory cytokines (IL-1β, IL-6, TNF-α and IFN-γ) and endotoxins can induce the expression of FB (3, 30, 34, 35). FB is an acute phase reactant, with an average increase of ~50% in plasma soon after infection (30, 34, 35).

**Figure 2:**
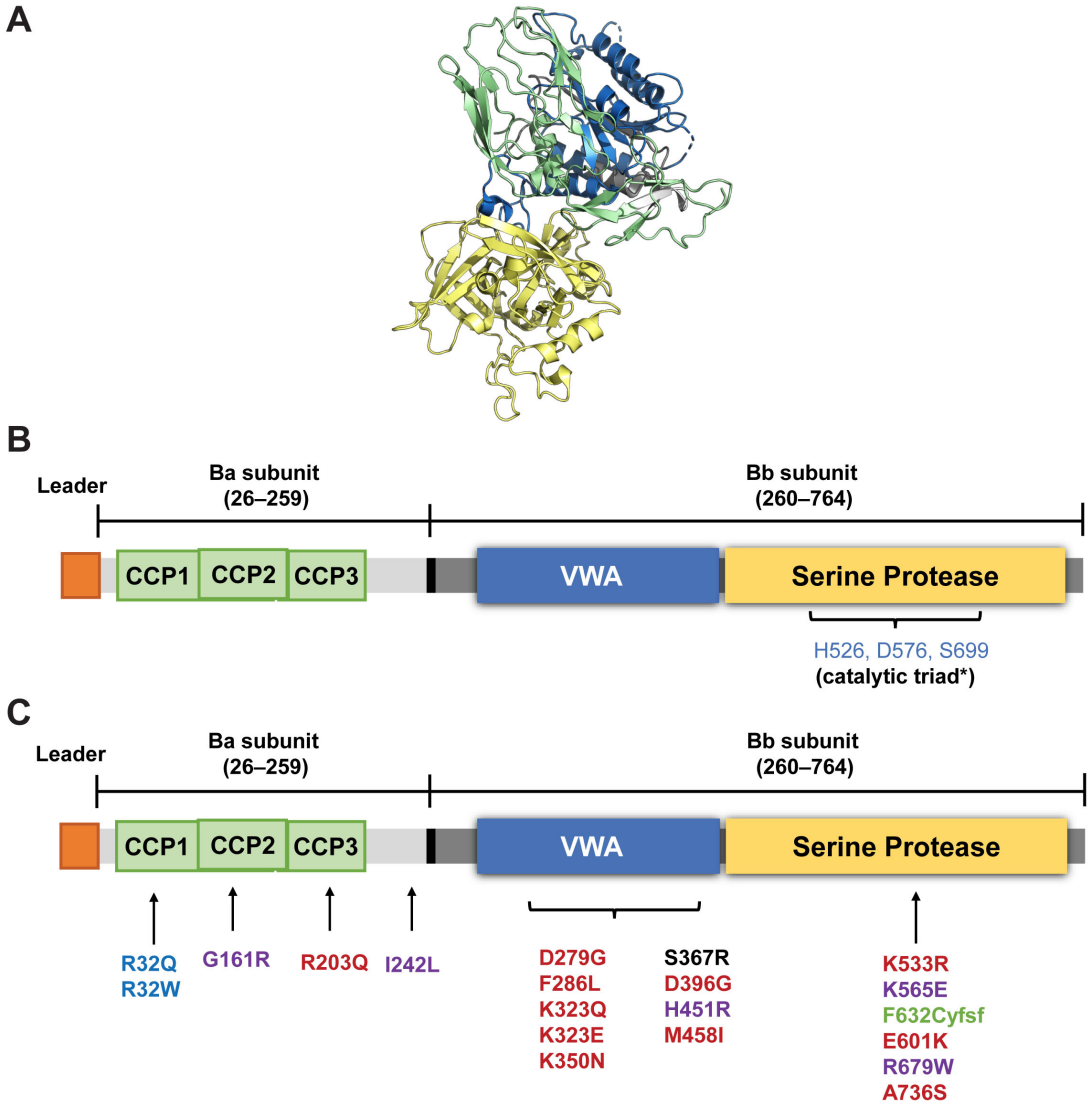
Structure of FB **(A)** and a schematic representation of its domains **(B, C)** Location of various polymorphic variants in FB reported in aHUS (red), C3G (black); MPGN (purple), and deficiency of FB (green). Slow-fast polymorphism variants R32Q (fast A) associated with reduced risk of age-related macular degeneration) and R32W (fast B) are in blue. *Iptacopan binds to the active site of FB containing the catalytic triad (HIS 526, Ser 699 and Asp576) in the serine protease domain of the Bb subunit (numbering according to UniProt (P00751). (Image **A** from RCSB PDB of 2OK5 created using Pymol: an open-source molecular visualization system. Figure **B** and **C** adapted with permission from Laskowski J, Thurman JM. Chapter 14 - Factor B. The Complement FactsBook: Academic Press; 2018. p. 135-46. Copyright 2018 by Elsevier) (30). CCP, complement control protein module; VWA, von Willebrand factor type A domain.

FB is a single-chain glycoprotein of 764 amino acids (~93kDa) consisting of two subunits: the non-catalytic N-terminal Ba subunit (~33 kDa; residues 26-259) linked to the C-terminal catalytic Bb subunit (~60 kDa, residues 260-764) via a 45–amino acid linker peptide encompassing the scissile bond (Arg 259-Lys260) that is cleaved by FD (**Figures 2A, B**) (30). The N-terminal Ba subunit consists of three complement control protein (CCP) modules or short consensus repeat domains of ~60 amino acids each (**Figure 2B**) that are common to several proteins of the complement system. The Bb subunit consists of a von Willebrand factor type A domain along with the MIDAS (metal-ion dependent adhesion site) motif and the serine protease domain (3, 30).

**Figure 3:**
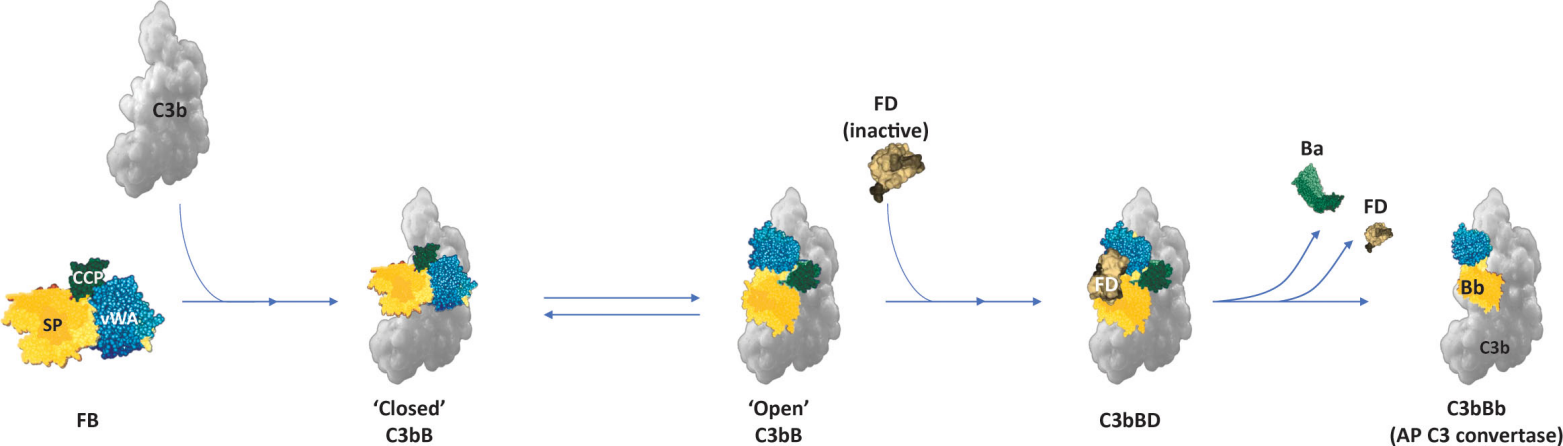
AP C3 convertase (C3bBb) formation. Binding of FB to C3b results in conformational changes in FB, with the serine protease domain dynamically oscillating between a “closed” and “open” form that allows the binding of FD and cleavage of the scissile bond to liberate the Ba fragment (1, 30). (Figure adapted from Merle NS, Church SE, Fremeaux-Bacchi V, Roumenina LT. Complement System Part I - Molecular Mechanisms of Activation and Regulation. Front Immunol. 2015;6:262, doi: 10.3389/fimmu.2015.00262. Copyright 2015 Merle, Church, Fremeaux-Bacchi and Roumenina. CC BY 4.0) (1). AP, alternative pathway; FB, Factor B; FD, Factor D.

FB circulates in the plasma in a latent zymogen form. To prevent indiscriminate activation of FB, the Ba subunit folds back onto the Bb subunit, thus preventing binding of C3b [or C3(H2O)] with the MIDAS motif and preventing FD from accessing the scissile bond in unbound FB (36). Initial Mg^2+^-independent binding of C3b or [C3(H2O)] with the CCP domain of Ba subunits results in a conformational change, exposing the MIDAS motif. This allows the binding and stabilization of interaction between C3b [or C3(H2O)] and Bb subunit in an Mg^2+^-dependent manner and brings in another conformational change in FB that exposes the scissile bond in the linker region for cleavage by FD (**Figure 3**) (3, 30, 37). The FD–C3bB interaction induces a conformational change in FD, displacing its self-inhibitory loop (5, 38). This co-factor–dependent and substrate-induced proteolytic step is one of the regulatory mechanisms that restricts the uncontrolled activation and amplification of the AP on C3b-opsonized surfaces (38). FD cleaves the linker peptide at the scissile bond, generating the Ba fragment that dissociates from the complex, while Bb with its serine protease domain remains associated with C3b to become the catalytically active component of the newly formed AP C3 convertase (**Figure 3**) (3, 5).

For spontaneous AP activation started by C3 hydrolysis [C3(H2O)], an additional FD-independent mechanism may exist (39). With the formation of convertase, the serine protease domain in Bb re-orients and becomes catalytically active. The C3 convertase cleaves C3 at Arg726-Ser727 in the C3 α chain; the C5 convertase cleaves C5 at homologous Arg74-Leu75 in the C5 α chain (30).

C3 and C5 are the only known natural substrates of the convertases (C3bBb and C3bBbC3b, respectively). Both the initial AP fluid-phase [C3(H2O)Bb] and the C3bBb C3 convertases alone have very short half-lives (~90 seconds for C3bBb) and rapidly dissociate (3). The Bb fragment has only marginal catalytic activity outside of the C3bBb complex, and once Bb dissociates from the convertase complex, it cannot reassociate with C3b (3, 5).

The stability of the AP convertases can be affected by its interactome. Interaction with properdin can increase the stability of the AP C3 convertase (C3bBb) up to 10-fold on target cell surfaces as it prevents C3 convertase dissociation by Factor H (FH) and cleavage by Factor (FI), thus enhancing complement amplification. Negative regulators, such as FH, FI, membrane cofactor protein (MCP), complement receptor 1 (CR1), and decay accelerating factor (DAF), down-regulate the AP by facilitating AP convertase dissociation (1, 3, 5, 30, 40–43).

FH is the master regulator of the AP; it blocks the amplification loop both in the fluid phase and on surfaces by competitively inhibiting the interaction of FB with C3b, thus preventing the formation of new C3 convertase (1, 40, 41, 44). FH also controls the AP by promoting accelerated dissociation of the AP C3 convertases to C3b and Bb (decay-accelerating activity) and by acting as a cofactor for FI, which cleaves and deactivates C3b to iC3b on cell surfaces. DAF, MCP, and CR1 are membrane-bound proteins and contribute to the regulation of all three complement pathways (1, 40, 41).

### 2.1 FB deficiency and variants

FB deficiency is very rare, with only three reported cases (45–47). As with FD deficiency, a deficiency of FB is associated with an increased risk of encapsulated bacterial infections, including meningococcal infections.

Three major allelic variants of *CFB* (differing at nucleotide position 32 [amino acid 7] of the Ba domain), collectively referred to as the slow-fast polymorphism (based on electrophoretic mobility), have been described as “slow” A, “fast” A, and “fast” B. These variants have been shown to have an effect on complement activation. Compared to the common allele, the “slow” variant (32R) of *CFB*, the “fast” A variant (32Q) and “fast” B variant (32W) are less efficient in AP C3 convertase (C3bBb) formation. The “fast” A variant displays a disease-protective effect against AMD (30, 48). Several rare variants of FB have been associated with complement-mediated diseases such as aHUS, C3G (rarely), MPGN, and AMD (mainly gain-of-function, [GoF] variants), with some variants (loss-of-function [LoF] variants) conferring protection against developing AMD (**Figure 2C**) (30, 49–53).

Similarly, mice deficient in *CFB* develop normally but lack AP activity and are more susceptible to bacterial infections (54, 55). Deletion of *CFB* (−/−) has been shown to rescue the renal phenotype in *CFH* KO mice (that exhibit a C3G-like pathology due to uncontrolled AP activation) and leads to a less severe renal disease and immune complex deposition in LN disease models, highlighting the critical role of FB in driving the AP/amplification loop and in the pathophysiology of complement-mediated diseases (55–57).

## 3 Targeting FB in complement-mediated diseases

**Figure 4:**
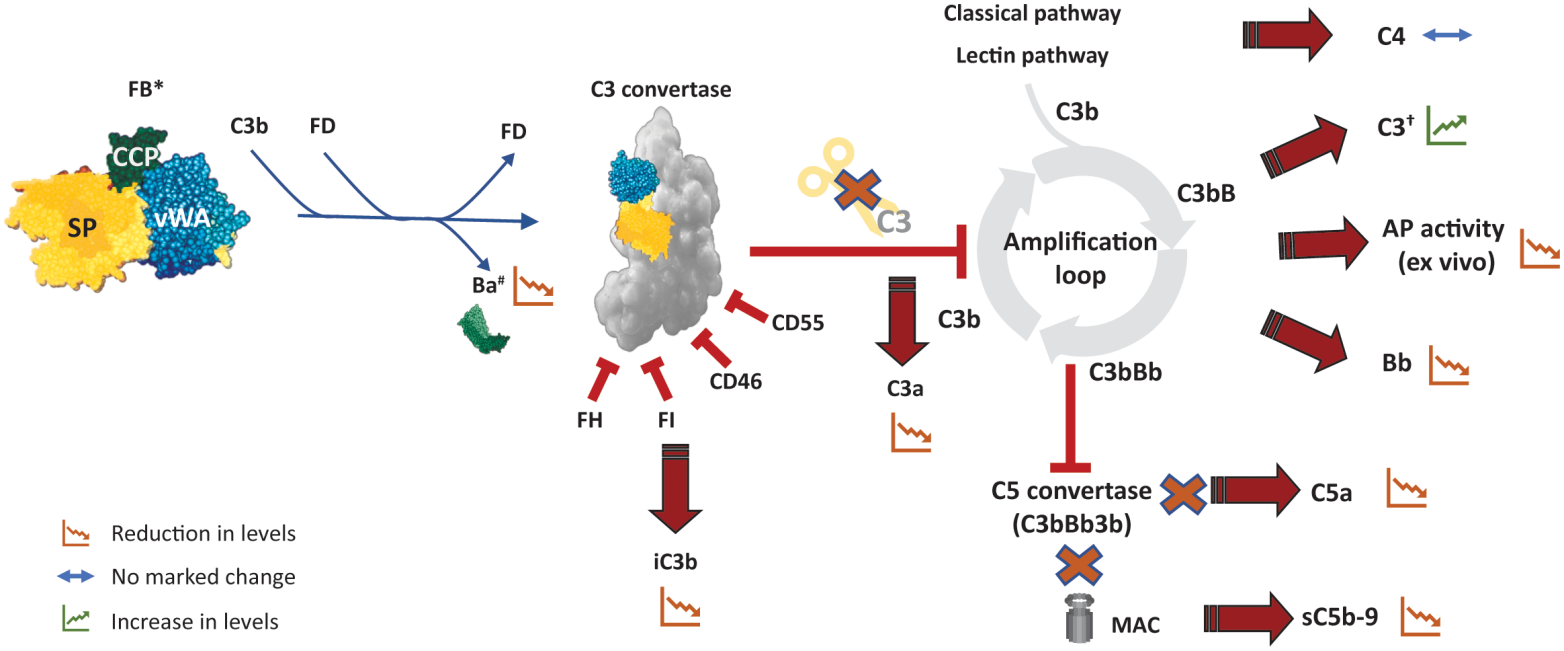
Potential pharmacodynamic effects of FB inhibition on complement activation. The effect of an inhibitor that inhibits catalytic activity of FB (and hence of C3/C5 convertase) is depicted here. Inhibition of FB abolishes cleavage of C3 by the AP C3 convertase and eventually inhibits the amplification loop as well as the downstream formation of AP C5 convertase. *An ASO (or siRNA) targeting FB would lead to reduction in the levels of circulating FB and its fragments in addition to the other effects described here. ^†^Particularly likely to be evident in conditions that result in C3 hypocomplementemia such as C3G. AP, alternative pathway, ASO, antisense oligonucleotide; C3G, C3 glomerulopathy; DAF, decay accelerating factor; FB, factor B; FD, factor D; FH, factor H; MCP, membrane cofactor protein; sC5b-9, soluble C5b-9; siRNA, small interfering RNA.

With increased knowledge of complement biology and its key role in the pathogenesis of several diseases, the complement system, and in particular the AP, has emerged as an innovative therapeutic target. Inhibition of the AP may be achieved either by preventing the formation of the new C3bBb complexes or by inhibiting the catalytic activity of the convertase (3, 5). Specific inhibition of the AP prevents formation of C3a and opsonization by C3b, halting the subsequent inflammation and phagocytosis of the opsonized cells (**Figure 4**), which are not impacted by distal inhibition of the complement pathway (e.g., at the level of C5). Several novel pharmacological agents targeting diverse aspects of the complement system are at various stages of clinical evaluation. Among potential targets that can specifically inhibit the AP and the amplification loop, FB, owing to its biology, has emerged as a promising pharmacological drug target (**Table 1**). FB is one of the upstream, proximal components unique to the AP and is essential for the activation of the pathway; it is an integral component that drives the amplification loop. FB has a relatively high plasma concentration; however, the plasma half-life of FB ranges from 30 to 70 hours, and it has fractional metabolic rates of 1.6%/h, allowing steady-state and stable inhibition (3). In comparison, FD inhibition might be more challenging than initially thought due to its rapid turn-over rate and self-inhibitory loop that may hinder the access of low-molecular-weight (LMW) inhibitors to the active site. Even a small amount of residual functional FD (1%-2%) is sufficient for restoring partial AP activity (11). This necessitates almost complete saturation of FD for complete and sustained inhibition (5, 6). While plasma FD levels are normally very low, they strongly increase in renally impaired individuals (10). Importantly, AP blockade (with FB and FD) inhibitors would also prevent amplification of the CP and LP (6). The anticipated effects of FB inhibitors on the pharmacodynamics of biomarkers of complement activation are summarized in **Figure 4**.

**Table 1:** A comparison of the various classes of complement inhibitors impacting the AP and terminal complement pathways.

	Factor B inhibitors	Factor D inhibitors	MASP-3 inhibitors	C3 inhibitors	C5 inhibitors	C5a/C5aR inhibitors
**Target**	FB is an upstream, proximal protease, specific to AP	FD is an upstream, proximal protease of AP	MASP-3 is an upstream protease activator of pro-FD	C3 is the central component of all complement pathways, not specific to AP	C5 component of the downstream, distal terminal pathway	C5a component of the downstream, distal terminal pathway
**Mode of action**	Inhibits the activity of AP C3 and C5 convertases, preventing cleavage of C3 and C5	Inhibits the cleavage of FB by FD and formation of functional AP convertases	Inhibits the activity of MASP-3, preventing the maturation/conversion of pro-FD to FD	Inhibits the cleavage of C3 into C3a and C3b	Inhibits the cleavage of C5 into C5a and C5b and subsequent MAC formation	Inhibits C5a/C5a receptor to block the biological effects of C5a. C5aR antagonists may be specific for C5aR1, which mediates pro-inflammatory effects of C5a
**Effect on C3 cleavage and opsonization with C3 fragments**	Yes	Yes	Yes	Yes	No	No
**Direct/indirect effect on C5 cleavage**	Yes	Yes	Yes	Yes	Yes	No
**Effect on other complement pathways**	Specific inhibition of the AP and amplification loop; does not inhibit direct activation of the CP or LP	Specific inhibition of the AP and amplification loop; does not inhibit direct activation of the CP or LP	Specific inhibition of the AP and the amplification loop; does not inhibit direct activation of the CP or LP	Complete inhibition of all three pathways	Complete inhibition of terminal pathway triggered by all three pathways	Inhibits the effects of C5a generated by all three pathways
**MAC formation**	Blocks the formation of MAC triggered by the AP and amplification loop	Blocks the formation of MAC triggered by the AP and amplification loop	Blocks the formation of MAC triggered by the AP and amplification loop	Blocks the formation of MAC triggered by all three pathways	Prevents MAC formation by all three pathways	Does not affect MAC formation
**Serum/Plasma concentration of target protein**	0.2 mg/mL, with long half-life; levels largely independent of kidney function	0.001 mg/mL, high turn-over rate; cleared through kidney; impaired kidney function leads to elevated levels of FD	Mean serum concentration 6.4 mg/L (range: 2-12.9 mg/L), i.e., 0.0064 mg/mL; found in complex with Ficolin-3 (102)	1.2 to ~2 mg/mL (16, 103); a daily turnover of 40%	0.075 mg/mL (104)	(<120 pg/mL i.e., 1.2 × 10^-7^ mg/mL) (105)
**Risk of encapsulated bacterial infections**	Risk of infection with encapsulated bacteria; may be mitigated by vaccinations (16)	Risk of infection with encapsulated bacteria; may be mitigated by vaccinations (16)	Risk of infection with encapsulated bacteria; may be mitigated by vaccinations	Higher risk of infection due to broader inhibition of the complement system	Increased risk of Neisseria infections; unlikely to be mitigated by vaccinations (106)	Lower risk of encapsulated bacterial infections as C5a inhibition does not affect MAC formation

AP, alternative pathway; CP, classical pathway; FB, factor B; FD, factor D; MAC, membrane attack complex; MASP- mannose-binding lectin-associated serine proteases-3.

## 4 FB inhibitors currently in clinical development

Development of selective FB inhibitors was considered challenging primarily because high drug levels would be needed for efficient inhibition of the high serum levels of FB. Nevertheless, multiple compounds targeting FB are currently in various stages of development. These range from highly selective small-molecule inhibitors to antisense oligonucleotides and antibodies; the efficacy and safety of a few of these are being evaluated in ongoing clinical trials.

### 4.1 Iptacopan (Fabhalta^®^)

**Figure 5:**
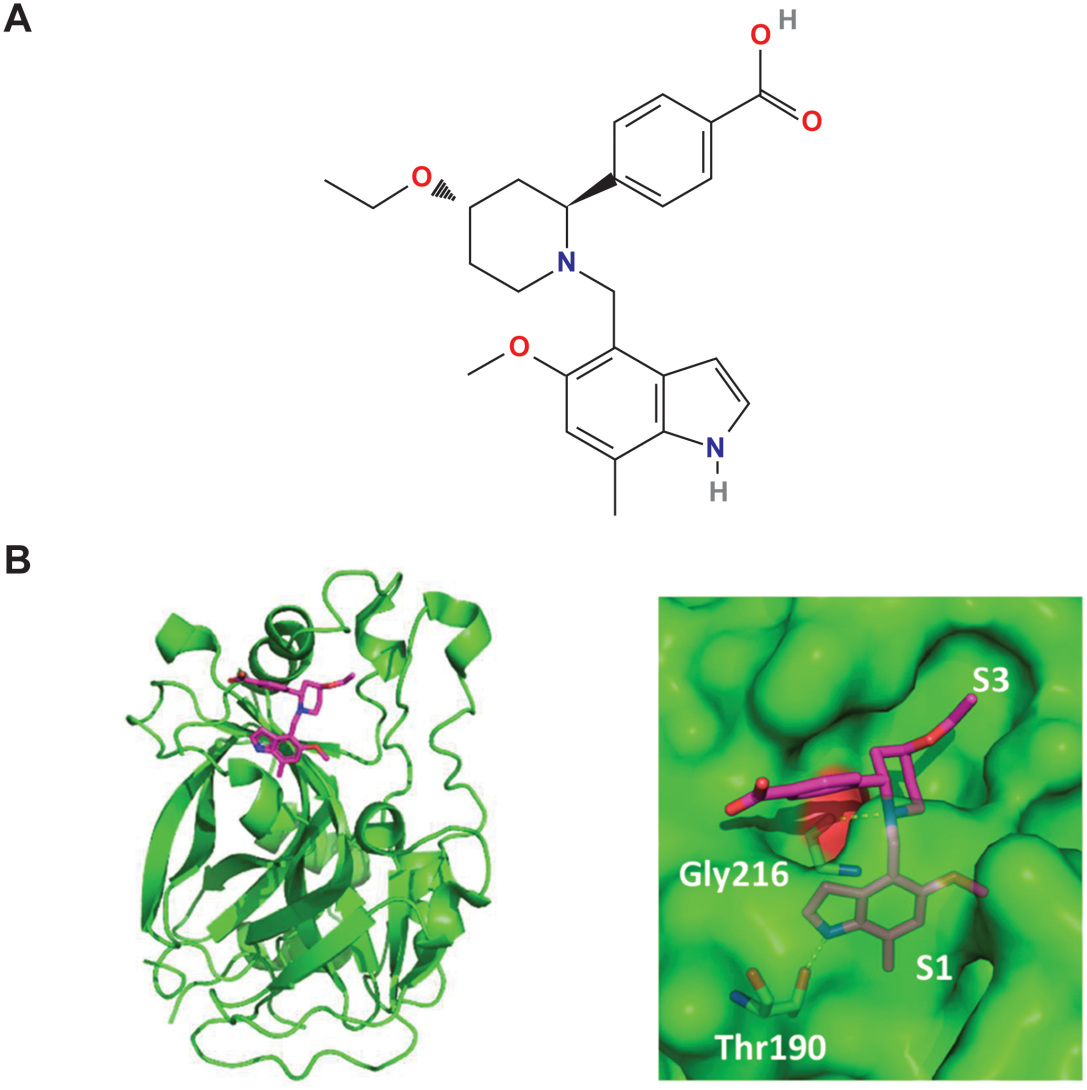
**(A)** Chemical structure of iptacopan (Figure **A** reprinted from PubChem. https://pubchem.ncbi.nlm.nih.gov/compound/lnp023. In public domain) and **(B)** Crystal structure showing iptacopan (magenta) bound to the catalytic domain of human FB. The image on the right shows the indole moiety and the piperidine core of iptacopan binding into the S1 pocket and the S3 pocket, respectively; and the key hydrogen bonds. (Figure **B** reprinted from Schubart A et al. Immunol Rev. 2023; 313(1):339-357, doi: 10.1111/imr.v313. Copyrights 2022 Novartis Pharma AG. Immunological Reviews published by John Wiley & Sons Ltd. CC BY-NC-ND 4.0) (3).

Iptacopan (Novartis Pharma AG) is an oral, proximal complement inhibitor that specifically binds FB and inhibits AP activity. Iptacopan was approved by the US FDA as the first oral monotherapy for adults with PNH in December 2023 and is the first complement inhibitor to receive accelerated approval for reduction of proteinuria in adults with primary IgAN who are at risk of disease progression (i.e. urine protein to creatinine ratio ≥1.5 g/g) (27, 29, 58). It is also being evaluated as a potential therapy in several other complement-mediated diseases such as C3G, aHUS, IC-MPGN, LN, and AMD (**Table 2**). Iptacopan binds to the active site of FB present in the Bb subunit (both to the native FB and the Bb fragment attached to C3b) with high affinity (KD value 0.0079 ± 0.0019 μM) and is highly selective over all proteases tested, including FD and CP/LP activity (**Figure 5**) (3, 59).

**Table 2:** Phase 2 and phase 3 clinical trials* of FB inhibitors.

Compound	Indication	Comparator	Phase 2/3	Clinicaltrials.gov identifier
**Iptacopan**	PNH (28, 62, 63)	Add-on to anti-C5 antibody	Phase 2	NCT03439839 (completed)
		None (treatment naive)	Phase 2	NCT03896152 (completed)
		Anti-C5 antibody	Phase 3	NCT04558918 (APPLY-PNH; completed)
		None	Phase 3	NCT04820530 (APPOINT-PNH; completed)
	IgAN (61, 68)	Placebo	Phase 2	NCT03373461 (completed)
		Placebo	Phase 3	NCT04578834 (APPLAUSE-IgAN; active, not recruiting)
			Roll-over extension	NCT04557462 (recruiting)
	C3G (60, 67)	None	Phase 2	NCT03832114 (completed)
		Placebo	Phase 3	NCT04817618 (APPEAR-C3G; recruiting)
	aHUS (66, 70, 107)	None	Phase 3	NCT04889430 (APPELHUS; recruiting)
		None	Phase 3	NCT05935215 (switch study; recruiting)
	IC-MPGN (65)	Placebo	Phase 3	NCT05755386 (APPARENT recruiting)
	LN (108)	Placebo	Phase 2	NCT05268289 (recruiting)
	AMD (109)	Placebo	Phase 2	NCT05230537 (recruiting)
	gMG (110)	Placebo	Phase 3	NCT06517758 (recruiting)
**IONIS-FB-LRx** (RO743465)	IgAN (111)	None	Phase 2	NCT04014335 (completed)
	IgAN (76)	Placebo	Phase 3	NCT05797610 (IMAGINATION; recruiting)
	AMD^#^ (112)	Placebo	Phase 2	NCT03815825 (GOLDEN; completed)
	AMD^#^ (113)	Placebo	Phase 2	NCT03446144 (withdrawn)
**NM8074**	PNH (87–89)	None	Phase 2	NCT05646524 (not yet recruiting)
		None^†^	Phase2	NCT05646563 (not yet recruiting)
		None	Phase 2	NCT05731050 (not yet recruiting)
	aHUS (85)	None	Phase 2	NCT05684159 (not yet recruiting)
	C3G (86)	None	Phase 1/2	NCT05647811 (not yet recruiting)
HRS5965	PNH (93)	None	Phase 2	NCT06051357 (active, not recruiting)
	IgAN (77)	Placebo	Phase 2	NCT06137768 (not yet recruiting)
MY008211A	PNH (91, 92)	None	Phase 2	NCT06050226 (recruiting)
		None	Phase 2	NCT06134414 (not yet recruiting)

*Status as on December 1, 2024. # Roche has discontinued IONIS-FB-LRx AMD program in July 2024. ^†^NM8074 will be evaluated as a monotherapy as well as in combination with eculizumab.

aHUS, atypical hemolytic uremic syndrome; AMD, age-related macular degeneration; C3G, C3 glomerulopathy; CAD, cold agglutinin disease; IC-MPGN, immune-complex mediated membranoproliferative glomerulonephritis; IgAN, immunoglobulin A nephropathy; ITP, immune thrombocytopenia; gMG, myasthenia gravis; LN, lupus nephritis; PNH, paroxysmal nocturnal hemoglobinuria.

Upon oral administration, iptacopan is rapidly absorbed, reaching peak plasma concentration in ~2 hours. In phase 2 studies, iptacopan dose-dependently inhibited AP activity, with a maximal sustained inhibition at a dose of 200 mg twice daily (60, 61). Similarly, iptacopan treatment also resulted in a reduction of Bb levels, which is reflective of the extent of the AP activation, as well as of urinary sC5b-9 levels, reflecting the inhibition of the AP and terminal complement pathway within the kidney (61). In patients with C3G and reduced C3 (<77 mg/dL), normalization of mean plasma C3 levels was observed following iptacopan treatment for 3 months in most patients (60). Taken together, these results highlight that treatment with iptacopan effectively blocks the AP.

Evidence from PNH clinical trials and those emerging from other phase 2 and phase 3 trials support the efficacy of iptacopan in various complement-mediated diseases. Initially, iptacopan was tested as an add-on therapy to eculizumab and was well-tolerated. It led to significant improvement in hemoglobin and reduction in lactate dehydrogenase (LDH) and the effect was maintained even when anti-C5 treatment was discontinued (62). Consistent with these findings, in another small phase 2 study in treatment-naïve patients with PNH, iptacopan monotherapy was well-tolerated and led to a significant reduction in serum LDH levels along with transfusion-free improvements in hemoglobin levels in most patients (62, 63). Corroborating these observations are the results from the pivotal phase 3 studies in APPLY-PNH and APPOINT-PNH (28). In APPLY-PNH, a significantly higher proportion of patients treated with iptacopan monotherapy achieved hemoglobin increases of ≥2 g/dL (82% vs 2%; p<0.0001) and hemoglobin levels of ≥12 g/dL (69% vs 2%; p <0.001) without the need for transfusions at 24-weeks compared to anti-C5 therapies; similar outcomes were achieved in treatment-naïve patients in the APPOINT study (28).

In a phase 2 study in patients with C3G, 12 months of iptacopan treatment resulted in a 45% reduction in proteinuria in patients with native kidneys and significantly reduced glomerular C3 deposition in patients with transplanted kidneys, with stabilization of kidney function in both cohorts (60). Similarly, in a phase 2 study in IgAN, iptacopan resulted in a dose-dependent reduction in proteinuria, with a clinically meaningful and statistically significant 23% reduction achieved at 3 months with iptacopan 200 mg twice daily compared to placebo (61). Iptacopan was generally safe and well tolerated in all the clinical studies, with no serious infections reported. Primary analysis results from the ongoing phase 3 studies in C3G and IgAN have corroborated the phase 2 findings. In the APPEAR-C3G study, patients treated with iptacopan achieved a 35.1% reduction in proteinuria (p=0.0014) at 6 months when compared to placebo on top of supportive care (64). Similarly, in the pre-specified interim analysis of APPLAUSE-IgAN study, iptacopan demonstrated superiority to placebo in reducing proteinuria in patients with IgAN, with a 38.3% (p<0.0001) reduction at 9 months when compared to placebo on top of supportive care (58). Based on these findings, iptacopan was granted accelerated approval by the US FDA for IgAN in August 2024 (29). Iptacopan is currently being evaluated in phase 3 studies for IgAN (APPLAUSE-IgAN), C3G (APPEAR-C3G), aHUS (APPELHUS), and IC-MPGN (APPARENT) (65–71) (**Table 2**). These promising data further strengthen the rationale that pharmacological inhibition of the AP, and of FB in particular, is an innovative therapeutic strategy.

### 4.2 IONIS-FB-LRx (RO743465/RG6299)

IONIS-FB-LRx (Ionis Pharmaceuticals/Roche) is an antisense oligonucleotide (ASO) that targets *CFB* mRNA in hepatocytes to prevent the synthesis of FB protein in the liver. It is a 2’-O-methoxyethyl (2’MOE) second-generation ASO (20 bp) conjugated to an *N-*acetylgalactosamine (GalNAc) ligand. As the liver is the main site of synthesis for FB, the GalNac moiety targets the ASO for uptake by hepatocytes (72). In a mouse model of LN, reduction of FB levels with ASO was associated with significant improvements in renal pathology, reduced glomerular C3 deposition and proteinuria, and improved survival (73). In a phase 1 study, administration of multiple, 20-mg doses of ASO for 36 days resulted in a 72% reduction in plasma FB levels and attenuation of AP activity by 62% (74).

In patients with IgAN, IONIS-FB-LRx administered every 4 weeks for 6 months resulted in a 44% reduction in proteinuria at week 29 (75). A phase 3 trial to evaluate the efficacy and safety of IONIS-FB-LRx in patients with IgAN has been initiated (**Table 2**) (76).

### 4.3 Compounds in early development phase

Several other compounds with diverse modes of action are being evaluated as potential FB inhibitors, including small molecule inhibitors such as the oral tablets MY008211A (in PNH) and HRS-5965 (in PNH and IgAN) (77, 78), a recombinant fragment of human FB (79), FB28.4.2 monoclonal antibody (mAb) targeting the Ba subunit to prevent interaction of C3b with FB (80, 81), mAbs (NM8074; SAR44380) targeting FB or its subunits, RNA interference therapies (ARO-CFB, ADX-038) targeting hepatic expression of FB (82, 83), and a cyclic hendecapeptide (complin) that prevents the cleavage of FB by FD and of C2 by activated C1s to inhibit the AP and CP/LP (84). Of these, the humanized mAb NM8074 (NovelMed) which binds to the Bb subunit (but not to the latent FB) to prevent the activity of C3/C5 convertases has entered phase 2 clinical development for C3G, aHUS, and PNH (**Table 2**) (85–89). Interim analysis from first-in-human studies of NM0874 in 40 healthy individuals suggests that NM8074 is safe and well tolerated across the doses tested (0.3-20 mg/kg), and specifically inhibits the AP but not the CP (90). Phase 2 trials have also been planned or are underway for HRS5965 (PNH and IgAN) and MY008211A (PNH) (77, 91–93). Phase 1 trial of SAR443809 has been completed, and phase 1/2a trial of ARO-CFB (in complement mediated kidney disease/IgAN) has been initiated (94–96).

## 5 Future perspectives and conclusions

In the last few decades, a vast body of data has demonstrated the role of complement and, in particular, the AP as key mediators of pathology in several diseases. An increased understanding of complement and its role in the pathogenesis of specific diseases, coupled with technological advances and a favorable regulatory framework for novel drugs in rare diseases, has spurred interest in the development of complement-targeted therapies that aim to halt or slow disease progression. Given the central role of the AP in amplifying the initial complement response, the AP and amplification loop have emerged as a key target, with FD, MASP-3, and FB being the prime candidates for inhibition (3, 6).

Several properties of FB make it an ideal therapeutic target for AP inhibition, and diverse approaches have been employed to target FB, each with its own strengths and limitations:

- Developing small-molecule inhibitors of FB was considered to be challenging due to the high serum concentration of FB. with iptacopan being the only FB inhibitor approved for PNH and IgAN (accelerated approval) and is in late-stage clinical development for several diseases (3, 5). A small-molecule inhibitor with good oral bioavailability and pharmacokinetic/pharmacodynamic properties has several advantages: a convenient route of administration for patients, potential for complete or near-complete therapeutic inhibition without disrupting the steady-state levels of the target protein, and owing to a reasonably short half-life, normal AP activity can be restored relatively quickly when needed (e.g., during infection) (3, 5, 6). In diseases such as aHUS, a rapid inhibition of the complement system with an oral inhibitor, as observed with iptacopan (maximal inhibition of the AP observed 2 hours post-dose), may be particularly relevant as an alternative therapeutic option vis-à-vis current intravenously administered SoC. On the other hand, a shorter half-life of the compound compared to an antibody requires good patient adherence to avoid incomplete complement inhibition after washout, in particular in acute indications.
- An antibody-mediated approach is perhaps less challenging: the convertase presents as a large substrate to a bulky antibody that needs to prevent access to C3b or FD (to prevent convertase formation) or to C3 (to inhibit its catalytic activity). This relative ease is reflected in several candidate antibodies that are being or have been explored as FB inhibitors. However, the bulky nature of antibodies necessitates an intravenous or subcutaneous mode of administration, Patients with aHUS report the need for chronic biweekly (or bimonthly) administration of anti-C5 antibodies as a significant burden (6, 26); estimated infusion duration is about 35 minutes to 1.5 hours (depending on body weight), and patients need to be under observation for 1 hour thereafter to monitor for infusion-related reactions (97, 98). In addition, the high plasma concentration of FB, as well as the increase in the half-life of targets of therapeutic antibodies, necessitate higher doses of antibodies to sustain FB inhibition.
- An ASO-based targeted approach to inhibit hepatic synthesis of FB is being evaluated/spearheaded by Ionis/Roche. However, similar to antibody-based approaches, ASOs would need chronic subcutaneous injections every month; moreover, ASO therapies may not be convenient in an acute setting since FB reduction occurs after a few days (74). In addition, whether inhibiting hepatic synthesis of FB with ASOs would have beneficial effects in instances where FB production occurs locally at the site of injury/inflammation remains to be elucidated.

Most of the current complement-targeted therapies, including those targeting FB, have been or are currently being evaluated in prototypical complement-mediated kidney diseases where the AP drives pathogenesis and mediates tissue injury. In addition to these, FB inhibitors may also have a beneficial role in diseases where AP activation may be secondary to other triggers (3, 6). For example, in multiple autoimmune diseases, such as in LN where autoantibodies initially trigger the CP, the amplification loop is expected to subsequently contribute to the pathology.

Although most of the complement-mediated therapies in development or already in use have generally been safe, the increased risk of infection with encapsulated bacteria remains a safety concern for this class of drugs, given the important role the complement system plays in fighting invading pathogens. In general, the risk of severe infection, especially Neisseria infections, is expected to be higher with more distal complement inhibitors (such as anti-C5 therapies). Inhibition of C3 is also expected to be typically associated with the risk of a broader class of infections. Mitigation strategies such as vaccination against encapsulated bacteria and prophylactic antibiotics where needed are essential to reduce the risk of infections. Indeed, sera from vaccinated individuals treated with AP inhibitors retained higher bactericidal and opsonophagocytic activity against encapsulated bacteria compared to those treated with C5 or C3 inhibitors *in vitro* (16–18, 99). Such mitigation strategies have also been proposed as part of the design and conduct of clinical trials of complement-mediated therapies. Long-term, close safety monitoring for infections is nevertheless essential for patients being treated or considered for treatment with complement-targeted therapies (100).

In conclusion, AP blockade through FB inhibition is a promising addition to the armamentarium of potential therapies in complement-mediated kidney diseases. With the approval of iptacopan for treatment of adults with PNH and IgAN for patients at risk of progression with UPCR ≥1.5 g/g (accelerated approval), and potential approval for some of the other FB-inhibiting novel therapies in sight, patients with these rare complement-mediated diseases may soon have access to effective, targeted therapies that offer a better safety profile than conventional immunosuppression.
