# Supplementary material for: Factor B as a therapeutic target for the treatment of complement-mediated diseases
Source: Front Immunol. 2025 Feb 14;16:1537974. doi: 10.3389/fimmu.2025.1537974 (PMC11868072; doi:10.3389/fimmu.2025.1537974)
Supplement: Supplementary file 1 [file Table1.docx]

**Factor B as a therapeutic target for the treatment of complement-mediated diseases**

**David Kavanagh^1*^, Jonathan Barratt^2^, Anna Schubart^3^, Nicholas J A Webb^4^, Matthias Meier^4^, Fadi Fakhouri^5^**

^1^National Renal Complement Therapeutics Centre, Newcastle University, Newcastle-upon-Tyne, United Kingdom

^2^Department of Cardiovascular Sciences, University of Leicester, The John Walls Renal Unit, University Hospitals of Leicester NHS Trust, Leicester, United Kingdom

^3^Department of Immunology, Novartis BioMedical Research, Basel, Switzerland

^4^Novartis Pharma AG, Basel, Switzerland

^5^Service of Nephrology and Hypertension, Centre Hospitalier Universitaire Vaudois, UNIL, Lausanne, Switzerland

*** Correspondence:**David Kavanagh

National Renal Complement Therapeutics Centre,

Newcastle-upon-Tyne, Newcastle University, United Kingdom

e-mail : [david.kavanagh@newcastle.ac.uk](mailto:david.kavanagh@newcastle.ac.uk)

Telephone : 0191 282 5094

Supplementary Material

# Supplementary Table

**1.1 Supplementary Table 1:** An overview of a few diseases involving the alternative complement pathway and the respective therapies

| **Disease** | | **Key clinical features and pathophysiology** | **Role of the complement system** | **Current SoC and limitations** | **FDA approved complement-targeted therapies** |
| --- | --- | --- | --- | --- | --- |
| PNH (1, 2) | | Rare hematological disease characterized by a triad of intravascular hemolysis, thrombophilia, and bone-marrow failure. May lead to CKD (65% of cases)  Caused by clonal expansion of hematopoietic stem cells that carry somatic mutation in the *PIGA* gene, leading to loss of GPI-anchored proteins from RBCs | Loss of GPI-anchored complement regulators CD55 (that accelerates the decay of C3 convertases) and CD59 (that prevents MAC assembly) from RBC surface, making PNH RBCs vulnerable to complement-mediated hemolysis | Anti-C5 (eculizumab and ravulizumab) therapy:  *Limitations*   - Lack of proximal inhibition by C5 inhibitors leads to opsonization of PNH RBCs and subsequent EVH - Pharmacokinetic breakthrough   Anti-C5 (eculizumab and ravulizumab) therapy and anti-C3 (pegcetacoplan) therapy  *Limitations:*   - Need for chronic IV administration or SC infusions - Increased risk of Neisseria infection | Ravulizumab Eculizumab  Pegcetacoplan  Iptacopan* |
| aHUS (3-5) | A life-threatening, ultra-rare form of thrombotic microangiopathy characterized by the triad of acute kidney injury, thrombocytopenia, and microangiopathic hemolytic anemia  Caused by dysregulation of AP on cell surfaces that leads to endothelial cell damage, which triggers a procoagulant and proinflammatory state that results in thrombosis and hemolysis | | LoF variants in AP regulators, such as F*H, FI, and MCP* (or anti-CFH autoantibodies that inhibit its function), or GoF variants in components of AP C3 convertase (*C3, CFB*) result in impaired AP regulation on endothelial cell surfaces | Anti-C5 therapy (eculizumab and ravulizumab)   - *Limitations:* - Need for chronic IV administration, and lack of consensus and guidelines on whether, and when, anti-C5 treatment can be discontinued - Increased risk of Neisseria infection | Ravulizumab and Eculizumab |
| C3G (6-8) | A chronic, rare primary glomerulonephritis defined by dominant or exclusive glomerular C3 deposition resulting from dysregulated AP activation in fluid phase (in >90% of patients)  Two distinct subtypes on EM: C3 glomerulonephritis and dense deposit disease | | Caused by dysregulation of the AP in the fluid phase either due to:   - - Autoantibodies against AP C3 and C5 convertases or against FB that stabilize the convertases or against FH that inhibit FH function   - Mutations, polymorphisms, or rearrangements in genes encoding complement proteins and/or their regulators (including GoF mutations in *C3, CFB* or LoF mutations in *CFH, CFI and MCP* or genomic rearrangements in *CFHR* locus) | No approved therapy  Treatment options limited to supportive care (with ACEi/ARB) and immunosuppression with MMF and corticosteroids and/or other immunosuppressants, despite lack of conclusive evidence for their benefit  *Limitations:* All immunosuppressive therapies are associated with substantial toxicity, and AEs, including increased risk of infections | None |
| IC-MPGN (9-10 | A rare complement-mediated kidney disease that may be idiopathic (primary), with some features overlapping with C3G or secondary to chronic infections, autoimmune disorders, or monoclonal gammopathies | | Similar to C3G, IC-MPGN is caused by genetic defects in and/or autoantibodies against components of the AP or their regulators  The mechanism leading to initial Ig deposition is not well understood, but this Ig deposition likely triggers additional classical pathway activation | No disease-specific therapies exist  Limited treatment options beyond supportive care; consist mostly of corticosteroids along with additional immunosuppressive therapies (MMF and/or cyclophosphamide depending on the severity of proteinuria and kidney function loss) | None |
| IgAN (9,11,12) | A rare, heterogenous autoimmune disease that is also the most common form of primary glomerulonephritis  Caused by autoantibodies against galactose-deficient IgA that leads to the formation and deposition of IgA-containing immune-complexes in the glomerular mesangium—a hallmark of IgAN  Multi-hit model proposed as pathogenic mechanism | | IgA-containing immune complexes can directly activate the AP (and LP) *in vitro*  An imbalance in FHR1, FHR5, and FH may increase AP activation and C3 cleavage in response to mesangial IgA1 deposition, leading to complement-dependent glomerular inflammation and injury | High-risk patients with persistent proteinuria (>1 g/d) are often managed with supportive care (ACEi/ARB) and corticosteroids, despite the uncertain benefit:risk profile of corticosteroids  Two drugs have received approval in 2024:  Delayed-release formulation of corticosteroid budesonide (nefecon) was the first drug to receive FDA approval for reducing the loss of kidney function in adults with IgAN at risk of disease progression  Sparsentan, an endothelin and angiotensin II receptor antagonist, has recently received approval to slow kidney function decline in adults with IgAN who are at risk of disease progression.  *Limitations:* Corticosteroid use is associated with several AEs, including hypercortisolism and increased risk of infections, among others which adversely impact patients’ QoL.  Endothelin receptor antagonist use has a risk of hepatotoxicity | Iptacopan* |
| Lupus nephritis (7, 13-14) | | Glomerulonephritis developing as a severe renal manifestation of the autoimmune disease systemic lupus erythematosus  Caused by inefficient clearing of nuclear antigens and production of autoantibodies against these | Deposition of preformed and/or in situ formation of autoantibody-containing immune complexes in subendothelial tissue triggers CP activation. This initial complement response is amplified by the AP and drives kidney damage  Mutations, polymorphisms, and deletions in CP components such as *C1, C2, and C4*. Rare pathogenic variants in *CFH* and *CFI* are also reported | Induction of remission with intense immunosuppressive therapy (corticosteroids, MPA, cyclophosphamide) followed by a less aggressive maintenance phase  Only 30% to 40% of patients achieve complete or partial remission with SoC  Chronic immunosuppression is associated with toxicity (such as infections, malignancies and bone marrow toxicities) that can lead to poor adherence | None |
| ANCA-associated vasculitis (7,15,16) | | Multisystem, autoimmune disease caused by autoantibodies against neutrophil cytoplasmic proteins (typically myeloperoxidase and proteinase 3)  Heterogenous group characterized by vascular inflammation of small blood vessels with pauci-immune glomerulonephritis | Degranulation of activated neutrophils and NETs can activate the AP, leading to generation of C5a  C5a can recruit and prime neutrophils at the site of inflammation, resulting in an amplification loop | Induction of remission with cyclophosphamide, or rituximab combined with corticosteroids  Avacopan as an adjunct treatment to standard therapy  *Limitations:* Substantial toxicity and risk of cancer with immunosuppressive therapies | Avacopan |
| Geographic atrophy secondary to AMD (17,18) | | A multifactorial ocular disease with multiple pathways (environmental stress, oxidative stress, changes in ECM, complement, and inflammasome activation among others) driving the pathogenesis | Disease-associated mutations/polymorphisms in complement genes: *CFH, CFHR4, CFB, CFI, C2, C3, C8, C9*  Deposits of complement cleavage products around subretinal drusen and MAC in choriocapillaris and ciliary septa | No effective disease-targeted treatment until recently— pegcetacoplan and avacincaptad pegol were approved in 2023  *Limitations:* Increased risk of endophthalmitis  Small risk of retinal vasculitis with pegcetacoplan | Pegcetacoplan,  avacincaptad pegol (C5 inhibitor) |

Iptacopan received accelerated approval for reduction of proteinuria in adults with primary IgAN who are at risk of disease progression (UPCR ≥1.5 g/g)

ACEi, angiotensin-converting enzyme inhibitor; AE, adverse event; aHUS, atypical hemolytic uremic syndrome; AMD, age-related macular degeneration; ANCA, anti-neutrophil cytoplasmic antibody; AP, alternative complement pathway; ARB, angiotensin receptor blocker; C3G, C3 glomerulopathy; CFB, complement factor B; CFH, complement factor H; CFHR, complement factor H related; CFI, complement factor I; CKD, chronic kidney disease; CP, classical pathway; ECM, extracellular matrix; EM, electron microscopy; EVH, extravascular hemolysis; FB, factor B, FDA, US Food and Drug Administration; FH, factor H, FHR, factor H-related; FI, factor I; GoF, gain of function; GPI, glycosylphosphatidylinositol; IC-MPGN, immune complex-mediate membranoproliferative glomerulonephritis; Ig, immunoglobulin; IgAN, IgA nephropathy; IV, intravenous; LP, lectin pathway; LoF, loss of function; MAC, membrane attack complex; MCP, membrane co-factor protein; MMF, mycophenolate mofetil; MPA, mycophenolic acid; NET, neutrophil extracellular trap; PIGA, phosphatidylinositol glycan class A; PNH, paroxysmal nocturnal hemoglobinuria; RBC, red blood corpuscle; QoL, quality of life; SC, subcutaneous; SoC, standard of care

References

1. Bodo I, Amine I, Boban A, Bumbea H, Kulagin A, Lukina E, et al. Complement Inhibition in Paroxysmal Nocturnal Hemoglobinuria (PNH): A Systematic Review and Expert Opinion from Central Europe on Special Patient Populations. Adv Ther. (2023);40(6):2752-72.
2. Lee JW, Brodsky RA, Nishimura JI, Kulasekararaj AG. The role of the alternative pathway in paroxysmal nocturnal hemoglobinuria and emerging treatments. Expert Rev Clin Pharmacol. (2022);15(7):851-61.
3. Fakhouri F, Zuber J, Fremeaux-Bacchi V, Loirat C. Haemolytic uraemic syndrome. Lancet. (2017);390(10095):681-96
4. Werion A, Rondeau E. Application of C5 inhibitors in glomerular diseases in 2021. Kidney Res Clin Pract. (2022);41(4):412-21.
5. Syed YY. Ravulizumab: A Review in Atypical Haemolytic Uraemic Syndrome. Drugs. (2021) ;81(5):587-59
6. Smith RJH, Appel GB, Blom AM, Cook HT, D'Agati VD, Fakhouri F, et al. C3 glomerulopathy - understanding a rare complement-driven renal disease. Nat Rev Nephrol. (2019);15(3):129-43
7. Kidney Disease: Improving Global Outcomes Glomerular Diseases Work G. KDIGO 2021 Clinical Practice Guideline for the Management of Glomerular Diseases. Kidney Int. (2021);100(4S):S1-S276.
8. Caravaca-Fontan F, Lucientes L, Cavero T, Praga M. Update on C3 Glomerulopathy: A Complement-Mediated Disease. Nephron. (2020);144(6):272-80.
9. Poppelaars F, Thurman JM. Complement-mediated kidney diseases. Mol Immunol. (2020);128:175-87.
10. Noris M, Daina E, Remuzzi G. Membranoproliferative glomerulonephritis: no longer the same disease and may need very different treatment. Nephrol Dial Transplant. (2023);38(2):283-290
11. Medjeral-Thomas NR, Cook HT, Pickering MC. Complement activation in IgA nephropathy. Semin Immunopathol. (2021);43(5):679-90.
12. Rizk DV, Maillard N, Julian BA, Knoppova B, Green TJ, Novak J, et al. The Emerging Role of Complement Proteins as a Target for Therapy of IgA Nephropathy. Front Immunol. (2019);10:504.
13. Macedo AC, Isaac L. Systemic Lupus Erythematosus and Deficiencies of Early Components of the Complement Classical Pathway. Front Immunol. (2016);7:55.
14. Amadei N, Baracho GV, Nudelman V, Bastos W, Florido MP, Isaac L. Inherited complete factor I deficiency associated with systemic lupus erythematosus, higher susceptibility to infection and low levels of factor H. Scand J Immunol. (2001);53(6):615-21.
15. Prendecki M, McAdoo SP. Targeting complement in ANCA-associated vasculitis: insights from ADVOCATE. Nat Rev Nephrol. (2021);17(7):439-40.
16. Chen M, Jayne DRW, Zhao MH. Complement in ANCA-associated vasculitis: mechanisms and implications for management. Nat Rev Nephrol. (2017);13(6):359-67.
17. Cabral de Guimaraes TA, Daich Varela M, Georgiou M, Michaelides M. Treatments for dry age-related macular degeneration: therapeutic avenues, clinical trials and future directions. Br J Ophthalmol. (2022);106(3):297-304.
18. Armento A, Ueffing M, Clark SJ. The complement system in age-related macular degeneration. Cell Mol Life Sci. (2021);78(10):4487-505.
